# French Views of Medical Reform

**Published:** 1846-03

**Authors:** 


*French Views of Medical Reform.—Congress of Medical Practitioners now sitting in Paris.*—"In France there has long existed an *inferior* class of practitioners, denominated Officiers de Santé. The members of the *superior* class all possess diplomas in Medicine or Surgery. The 'Doctors in Medicine,' and the 'Doctors in Surgery,' receive precisely similar educations, undergo the like examinations, and are equally eligible to fill all the public medical offices and appointments. These constitute one grade of practitioners in France ; the Officiers de Santé constitute the other or inferior order. Should the superior and inferior grades be maintained? That was the question which the Congress undertook to discuss ; when, with characteristic boldness, it was resolved, without a dissentient voice, that *the inferior ought to be abolished.* Thus the medical senators of France have announced, not only to the government of their own country, but to all others, that the legislature of every nation is bound to furnish the commuuity with one well-educated and thoroughly qualified class of medical practitioners ; and that a body of *inferior* acquirement, whether styled Officiers de Santé or Apothecaries, ought not to be recognised by the law. What will the manœuvring apothecaries of Blackfriars and Regent Street say to this decision?"—*London Lancet.*

